# Increased cardiac vagal tone in childhood-only, adolescent-only, and persistently antisocial teenagers: the mediating role of low heart rate

**DOI:** 10.1017/S0033291724000552

**Published:** 2024-03-12

**Authors:** Adrian Raine, Lia Brodrick, Dustin Pardini, J. Richard Jennings, Rebecca Waller

**Affiliations:** 1 Departments of Criminology, Psychiatry, and Psychology, University of Pennsylvania, Philadelphia, USA; 2 Department of Psychiatry, Perelman School of Medicine, University of Pennsylvania, Philadelphia, USA; 3 School of Criminology and Criminal Justice, Arizona State University, Tempe, USA; 4 Departments of Psychiatry and Psychology, University of Pittsburgh, Pittsburgh, USA; 5 Department of Psychology, University of Pennsylvania, Philadelphia, USA

**Keywords:** heart rate, parasympathetic, persistently antisocial, stress, vagal tone

## Abstract

**Background.:**

Cardiac vagal tone is an indicator of parasympathetic nervous system functioning, and there is increasing interest in its relation to antisocial behavior. It is unclear however whether antisocial individuals are characterized by increased or decreased vagal tone, and whether increased vagal tone is the source of the low heart rate frequently reported in antisocial populations.

**Methods.:**

Participants consisted of four groups of community-dwelling adolescent boys aged 15.7 years: (1) controls, (2) childhood-only antisocial, (3) adolescent-only antisocial, and (4) persistently antisocial. Heart rate and vagal tone were assessed in three different conditions: rest, cognitive stressor, and social stressor.

**Results.:**

All three antisocial groups had both lower resting heart rates and increased vagal tone compared to the low antisocial controls across all three conditions. Low heart rate partially mediated the relationship between vagal tone and antisocial behavior.

**Conclusions.:**

Results indicate that increased vagal tone and reduced heart rate are relatively broad risk factors for different developmental forms of antisocial behavior. Findings are the first to implicate vagal tone as an explanatory factor in understanding heart rate – antisocial behavior relationships. Future experimental work using non-invasive vagus nerve stimulation or heart rate variability biofeedback is needed to more systematically evaluate this conclusion.

## Introduction

For more than 30 years, research into low heart rate as a risk factor for antisocial behavior has been ongoing and accelerating. One early finding documented that antisocial adolescent boys were characterized by low resting heart rate, a finding that led us to hypothesize that low heart rate reflects a vagal coping response to mild stress (Raine & Venables, 1984). Vagal tone as currently measured defines parasympathetic nervous system activation specific to cardiac functioning and is evaluated using an index of respiratory sinus arrythmia (RSA) – the variability in heart rate in synchrony with respiration. Three central questions emanate from this early literature. First, do studies overall show that low resting heart rate characterizes antisocial individuals? Second, are antisocial individuals characterized by increased – or decreased – vagal tone? Third, do any group differences in vagal tone explain why antisocial individuals are characterized by low heart rate?

The first question was initially addressed in a meta-analysis of studies examining this relationship, showing that antisocial individuals have lower resting heart rate (Raine, 1993). Updated systematic reviews and meta-analyses during the 30 years since this initial review have further confirmed the low heart rate – antisocial behavior relationship (de Looff et al., 2022; Lorber, 2004; Ortiz & Raine, 2004; Portnoy & Farrington, 2015). One notable exception by Fanti et al. (2019) consists of a meta-analysis specifically of case-control studies of conduct disordered children which found non-significant effects for baseline heart rate across the five research studies included in the analysis, although the effect size (*d* = −0.33) was in the predicted direction and was also found to be significant in eight correlational studies (*d* = −0.14). The low heart rate – antisocial relationship has been established in large samples of both men (*N* = 710 264; Latvala, Kuja-Halkola, Almqvist, Larsson, and Lichtenstein, 2015) and women (*N* = 12 499; Oskarsson et al., in press), and has been documented in multiple countries throughout the world (de Looff et al., 2022; Portnoy & Farrington, 2015; Raine, 2002a, 2002b). Furthermore, the most recent meta-analysis on vagal tone and antisocial behavior did not find any moderating influence of age, gender, year of publication, design, analysis type, and sample size, leading to one of their major conclusions being that ‘resting heart rate remains the best replicated biological correlate of antisocial behavior.’ (de Looff et al., 2022; p. 557).

Regarding the second question, it is less clear whether or not increased vagal tone is reliably associated with increased antisocial behavior (Glenn & McCauley, 2019). A number of studies have found that *reduced* vagal tone is associated with increased antisocial behavior (Pang & Beauchaine, 2013; Sarrate-Costa, Lila, Comes-Fayos, Moya-Albiol, & Romero-Martinez, 2022). Similarly, one review of callous unemotional (CU) traits in children suggested that high CU scores are associated with reduced baseline vagal tone as measured by RSA (respiratory sinus arrythmia), while recognizing that only a handful of studies exist (Wagner & Waller, 2020). The meta-analysis of Fanti et al. (2019) in case–control designs of conduct disordered children *v.* control children also observed reduced task-related vagal tone in those with conduct disorder (*d* = −0.21), although in correlational studies of conduct disorder neither baseline (−0.06) nor task-related (*d* = 0.00) vagal tone resulted in significant associations. Nevertheless, there is a case to be made for reduced vagal tone in relation to increased antisocial behavior.

In contrast to this literature, other studies have reported *increased* resting and task-related vagal tone to be significantly associated with increased antisocial behavior (Byrd et al., 2022; Dietrich et al., 2007; Gao, Huang, & Li, 2017; Goulter, Kimonis, Denson, & Begg, 2019; Jimenez-Camargo, Lochman, & Sellbom, 2017; Scarpa, Fikretoglu, & Luscher, 2000; Zhang & Gao, 2015). The most recent meta-analysis of vagal tone and antisocial behavior reveals complex findings. Resting vagal tone (as measured by RSA) showed a negative association (*d* = −0.16, *p* = 0.20, *k* = 21), while vagal tone as measured by RMSSD (root mean square of successive differences) during task performance indicated a positive relationship, indicating *increased* vagal tone being associated with increased antisocial behavior (*d* = 0.36, *p* = 0.331, *k* = 18; de Looff et al., 2022), with increased vagal tone additionally being argued to be the most reliable autonomic correlate of psychopathy. Similarly, a systematic review of violence in romantic relationships concluded that high vagal tone was associated with increased relationship violence (Han, Baucom, Timmons, & Margolin, 2021). Overall, the main conclusion drawn from the extant literature to date is that findings on vagal tone and antisocial behavior are mixed (Glenn & McCauley, 2019; Zhang & Gao, 2015). Given the availability of interventions to increase vagal tone in clinical populations, including externalizing disorders (Zhu, Zhang, Zhou, Kendrick, & Zhao, 2022), the question of whether antisocial populations are characterized by decreased or increased vagal tone is of particular importance.

With regard to the third question regarding whether vagal tone differences can explain low heart rate in antisocial individuals, this issue has not been empirically addressed. In discussing the mixed findings on vagal tone, Zhang and Gao (2015) argued that because a low resting heart rate is associated with increased vagal tone, antisocial behavior should be associated with increased, not decreased, vagal tone. Early work on low resting heart rate theorized that testing in an unfamiliar environment represents a mildly stressful event (Raine & Venables, 1984); individuals with increased vagal tone would be prepared for disengagement from this potentially threatening situation, and through passive emotional withdrawal would be relatively insensitive to socializing punishments, with one consequence being a lowering of heart rate. A more recent study observed that children with both low heart rate and increased vagal tone have increased externalizing behavior (Dietrich et al., 2007), findings which support the confluence of increased vagal tone and low heart rate. Nevertheless, no study has empirically tested this vagal coping hypothesis by examining whether low heart rate mediates the vagal tone – antisocial behavior relationship.

The current study aims to address these three questions in a longitudinal study of antisocial behavior – the Pittsburgh Youth Study (Ahonen, Farrington, Pardini, & Stouthamer-Loeber, 2021). We built on our prior work which had established four distinct developmental groups derived from antisocial behavior measures collected from ages 7 to 17, with heart rate and vagal tone collected at age 17 (Raine et al., 2005). Based on conclusions of prior reviews, we hypothesized that low heart rate would characterize antisocial behavior. Given the prior mixed findings on vagal tone, we did not generate a specific directional hypothesis on this parasympathetic nervous system construct. Based on the passive vagal coping hypothesis, however, it was predicted that a low heart rate would mediate any increased vagal tone – high antisocial relationship. Regarding the influence of task condition, we evaluated contrasting predictions that arise from different viewpoints which would predict a significant group × task interaction. Again based on the vagal passive coping hypothesis and on the finding that two of the prior meta-analyses had found a stronger effect size for heart rate in negative affect conditions than in resting conditions (Ortiz & Raine, 2004; Raine, 1993), we hypothesized that a stronger effect would be observed in the stressful speech task compared to other conditions. In contrast, because the meta-analysis of de Looff et al. (2022) did not observe autonomic effects for speech tasks or for the continuous performance task, we tested the alternative prediction that findings for vagal tone would be weaker or non-existent for these two tasks. Because five prior meta-analyses have found robust support for the negative relationship between resting heart rate and antisocial behavior (de Looff et al., 2022; Lorber, 2004; Ortiz & Raine, 2004; Portnoy & Farrington, 2015; Raine, 1993), we hypothesized that findings would be observed in this resting condition for heart rate. We also examined whether these autonomic measures are more associated with those with persistently antisocial behavior throughout childhood and adolescence given that this sample may represent a more serious group who have been found to be more likely to present with neurocognitive deficits compared to others (Raine et al., 2005), or alternatively whether these cardiac measures are risk factors for all developmental forms of behavior.

## Method

### Participants

Participants were drawn from the Pittsburgh Youth Study (Ahonen et al., 2021). Full details of background characteristics and initial subject recruitment are given in Loeber, Farrington, Stouthamer-Loeber, and van Kammen (1998). Briefly, 868 grade 1 boys from public schools in Pittsburgh were assessed by caretakers, teachers, and the boys themselves on 21 serious antisocial behaviors. The 250 most antisocial boys were selected for further study, together with 253 boys randomly selected from the remainder, to make a total sample of 503.

Of the original sample of 503, 335 (66.6%) participated in a substudy on the biosocial bases of aggressive and violent behavior. Those participating did not differ from those not participating on background factors (Raine et al., 2005). Antisocial behavior and delinquency measures collected each year from children, parents, and teachers across ages 7–16 years were cluster analyzed (Raine et al., 2005). Four groups were identified: controls, childhood-only, adolescent-only, and persistently antisocial (see Raine et al., 2005). Controls (*N* = 156) remained stably low on antisocial behavior. The adolescent-only group (*N* = 68) started off at exactly the same level as the controls but progressed to significant levels of antisocial behavior problems by late adolescence. The childhood-only group (*N* = 57) started off with high levels of antisocial behavior up to age 11 but declined thereafter. The persistently antisocial group (*N* = 44) started off high and showed even higher levels of antisocial behavior during later adolescence. Further details and construct validity of group membership may be found in Raine et al. (2005). Further details of the composition of the clusters and clustering methodology are also given in the online Supplement.

These four groups provide the basis for the current analyses. Sample sizes with complete data in the current study are outlined in Table 1. Fifty-one cases were missing on covariates, heart rate, or vagal tone, resulting in a total sample of 284; there was no selective attrition amongst the four groups of boys (χ^2^ = 3.92, = 3, *p* = 0.27). Full written informed consent was obtained from the boys and their parents, and study protocols were approved by IRBs at both the University of Southern California and the University of Pittsburgh.

### Psychophysiological testing procedures

Heart rate at age 17 was measured continuously during a resting period (3 min), a social stressor task (4 min), and a cognitive task (8 min). Resting heart rate was found to be lowest at the end of experimental procedures when participants were told that all tasks had been completed. In the social stressor task, participants were instructed to spend 2 min thinking about the worst/most stressful thing that had ever happened to them, after which they described the event for 2 more minutes. To increase the level of stress, two research assistants present in the room made critical comments on the speech, and the speech was video-recorded. Only heart rate recorded during the first 2 min (the thinking period) was analyzed as speaking and associated body movements result in artifacts to both heart rate and vagal tone (Laborde, Mosley, & Thayer, 2017).

For the cognitive task, Version 4.08 of the degraded stimulus version of the Continuous Performance Task was administered (CPT, Nuechterlein, Parasuraman, and Jiang, 1983). Visually degraded numbers ranging from 0 to 9 were flashed on a computer screen (placed 1 meter from the participant in his line of vision) for 40 ms at the rate of one per second. The participants’ task was to press a response button on a Gravis joystick only when they saw the figure ‘0’. Targets had a 0.25 probability of occurrence. After 10 presentations of the target stimulus only, participants were given two practice blocks with 80 trials/block. Thereafter, 6 blocks with 80 trials in each block were presented, lasting 8 min.

### Cardiovascular recording and data reduction

Heart rate was recorded using a Grass Model 12 acquisition system. Sensor medics Ag/AgCl electrodes were placed below the right collarbone and below the left lower rib using Medi-Trace Conductivity gel. Respiration rate was measured using a strain gage placed around the chest in conjunction with a strain gage bridge transducer coupler and sampled at 5 Hz. The ECG signal was digitized at 256 Hz and stored for off-line processing. Heart rate levels were calculated by averaging IBIs (inter-beat-intervals) throughout each condition and converting values to b.p.m. (beats per minute).

Time between successive R-waves was stored and input together with respiration rate into the PSPAT software program which conducted artifact correction, spectral analysis of the cardiac data, and corrected for any non-stationarity in the data (Weber, Molenaar, & Van der Molen, 1992). Frequency domain characteristics were conducted to estimate heart rate variability (Weber et al., 1992). A test for stationarity was performed using cross-spectral analysis on artifact-corrected IBIs to determine the amplitude / power of respiratory sinus arrythmia employing the participants’ respiratory and cardiac signals. All participants had breathing rates within the frequently employed high-frequency range (0.15–0.30 Hz) of heart period variability. The heart period times series from each condition were linearly detrended and mean centered, with the time series tapered using a Hamming window (Gianaros, Van der Veen, & Jennings, 2004). Cross-spectral power estimates were then determined with a point process algorithm and log transformed prior to analysis. This spectral power, respiratory sinus arrhythmia estimate, was taken as the indicator of cardiac parasympathetic activity, with higher values indicating increased cardiac vagal tone.

### Covariates

Reviews of vagal tone have highlighted key variables that can act as potential confounds (Allen, Chambers, & Towers, 2007; Laborde et al., 2017). These consist of current antidepressant, antipsychotic, and anti-hypertensive medication usage (yes/no), caffeine use (units/week), alcohol use (units/week), nicotine use (cigarettes/week), age, gender, height, weight, and obesity (BMI). These variables were assessed during an induction interview. Physical fitness was also assessed by asking participants how often and for how long they engaged in the following activities: jogging, basketball, walking, tennis, soccer, swimming, weight-lifting, dancing, martial arts, boxing, and baseball, with scores summed to gain an overall measure of fitness.

### Statistical analyses

The ten potential covariates (see above) were tested as candidate confounds, with covariates retained if they had any significant association with any of the three vagal tone measures, including any marginally significant ( *p* < 0.10) relationships. Significant effects were obtained for fitness (*r* = 0.14, *p* = 0.021), caffeine (*r* = −0.12, *p* = 0.034), height (*r* = −0.18, *p* = 0.002), age (*r* = −0.13, *p* = 0.029), and antidepressant/antipsychotic/anti-hypertensive medication (*r* = 0.22, *p* < 0.001). These were all entered as covariates into a repeated measure multivariate analysis of variance (RM-MANOVA) with the three vagal tone measures (condition) as the dependent variable and the cluster grouping (four groups) as the independent variable. Mauchly’s test of sphericity tested the null hypothesis that the error covariance matrix or the orthonormalized transformation index is proportional to an identity matrix, with the Greenhouse-Geisser correction used to adjust for any lack of sphericity (Greenhouse & Geisser, 1959). Effect sizes were calculated using Cohen’s *d* (Cohen, 1988).

A test of reduced heart rate as a mediator of the vagal tone – antisocial behavior relationship was assessed using the PROCESS macro using a bootstrapping procedure which improves estimates of standard errors to identify any mediation effect (Hayes, 2012). The extent of any mediating effect was assessed using a two-step logistic regression in which the effect was estimated by calculating the % reduction in variance explained (using Nagelkerke *r*^2^) by vagal tone on antisocial grouping after controlling for heart rate. All analyses employed the above-mentioned covariates. Data are available from the first author upon reasonable request for the sole purpose of confirming findings.

## Results

### Antisocial behavior and heart rate level

Means and SDs for the four groups on the three heart rate measures are given in Table 1. Results of the RM-MANOVA revealed no main effect of condition, *F* = 1.13, df (1.78, 515.3), *p* = 0.32, and no interaction between group and condition, *F* = 0.87, df (5.35, 515.3), *p* = 0.50. The main effect of group was significant, *F* = 3.86, df (3, 289), *p* = 0.01.

**Figure 1.**
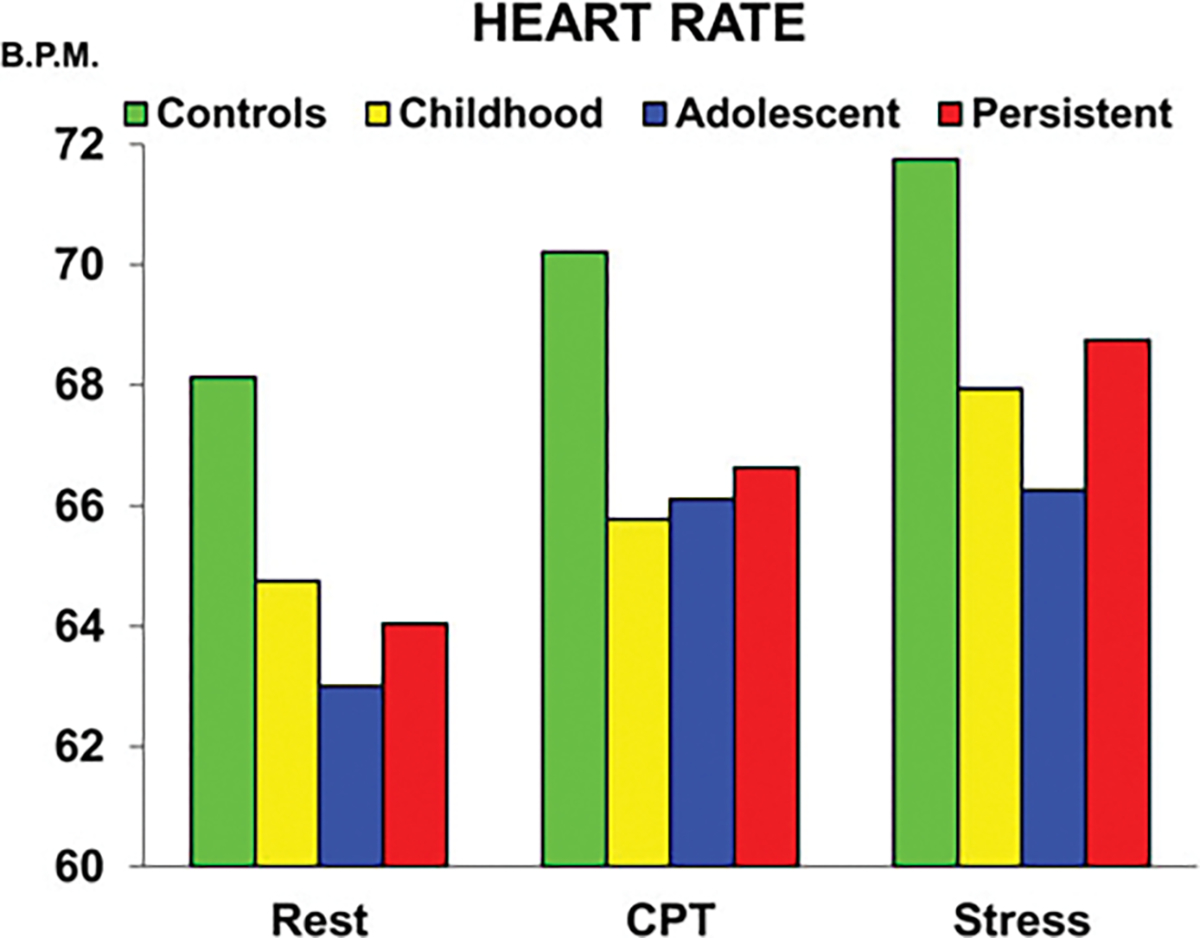
Heart rate levels in Controls, Childhood-Only, Adolescent-Only, and Persistently Antisocial groups during rest, cognitive stressor (CPT - continuous performance task) and social stressor conditions. All antisocial groups do not differ to one another, and all have lower heart rates than Controls.

Because no main effects of condition ( *p* = 0.32) or group × condition interaction ( *p* = 0.50) effects were observed, heart rate level was averaged across the three conditions and one-way ANOVA’s were conducted. Findings are illustrated in Fig. 1. There was a main effect of group, *F* = 3.86, df = 3289, *p* = 0.01. Compared to controls, childhood-only ( *p* = 0.01, *d* = −0.41), adolescent-only ( *p* = 0.013, *d* = −0.37), and persistently antisocial ( *p* = 0.005, *d* = −0.50) groups all had lower heart rate levels. No differences were observed between the three antisocial groups ( *p* > 0.47), and consequently for mediation analyses all three groups were combined to form a comparison of the antisocial group (*N* = 139) *v.* control group (*N* = 145).

### Antisocial behavior and vagal tone

Means and SDs for the four groups on the three vagal tone measures are given in Table 1. Results revealed no main effect of condition, *F* = 0.46, df (1.93, 540.8), *p* = 0.63, and no interaction between group and condition, *F* = 0.63, df (5.80, 540.8), *p* = 0.77. The main effect of group was significant, *F* =3.45, df (3, 280), *p* = 0.017.

**Figure 2.**
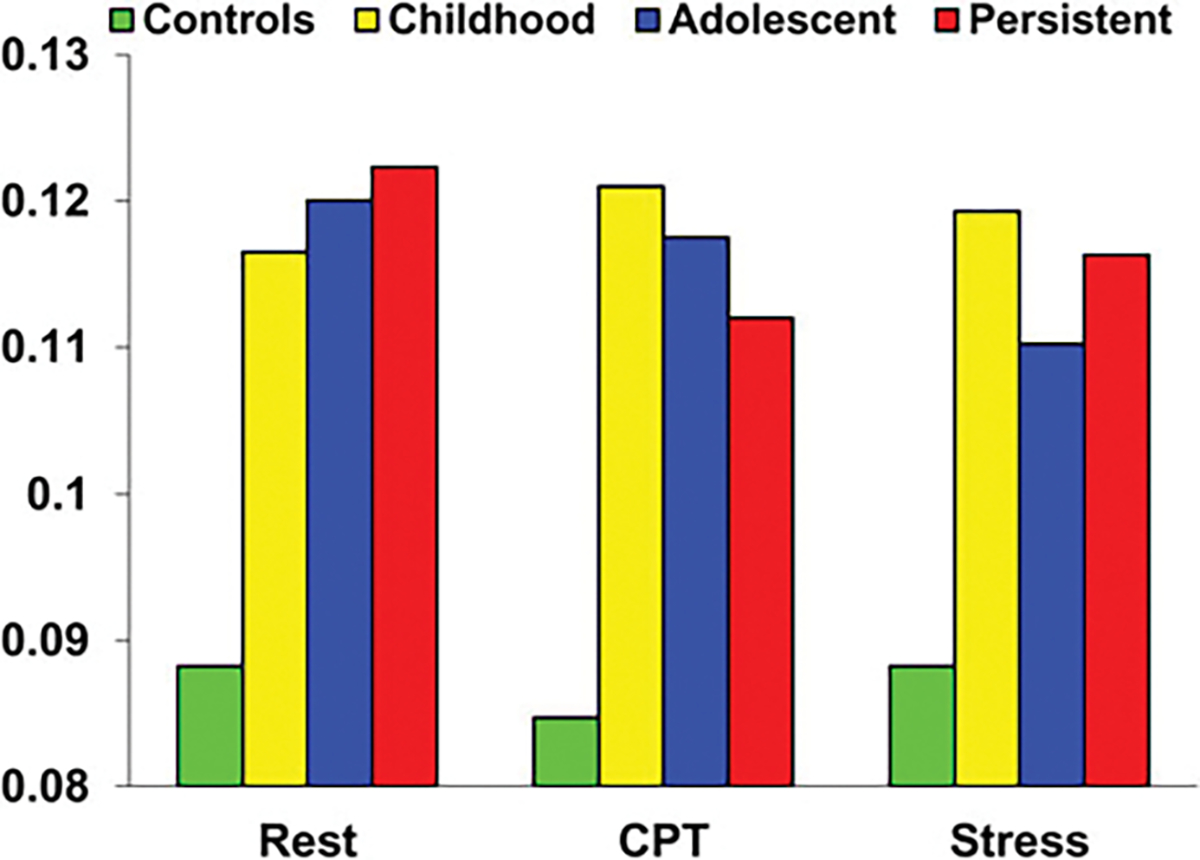
Vagal tone in Controls, Childhood-Only, Adolescent-Only, and Persistently Antisocial groups during rest, cognitive stressor (CPT - continuous performance task) and social stressor conditions. All three antisocial groups do not differ to one another, and all have higher vagal tone than Controls.

Because condition ( *p* = 0.63) and the group × condition interaction ( *p* = 0.63) effects were non-significant, vagal tone was averaged across the three conditions and one-way ANOVA’s conducted to further evaluate group differences. Findings are illustrated in Fig. 2. The main effect of group was significant, *F* = 3.45, df = 3280, *p* = 0.017. Compared to controls, childhood-only ( *p* = 0.021, d = 0.39), adolescent-only ( *p* = 0.009, *d* = 0.42), and persistently antisocial ( *p* = 0.008, *d* = 0.59) groups all had increased vagal tone. No significant differences were observed between the three antisocial groups ( *p* > 0.60), and consequently for mediation analyses all three groups were combined.

### Mediation analysis

**Figure 3.**
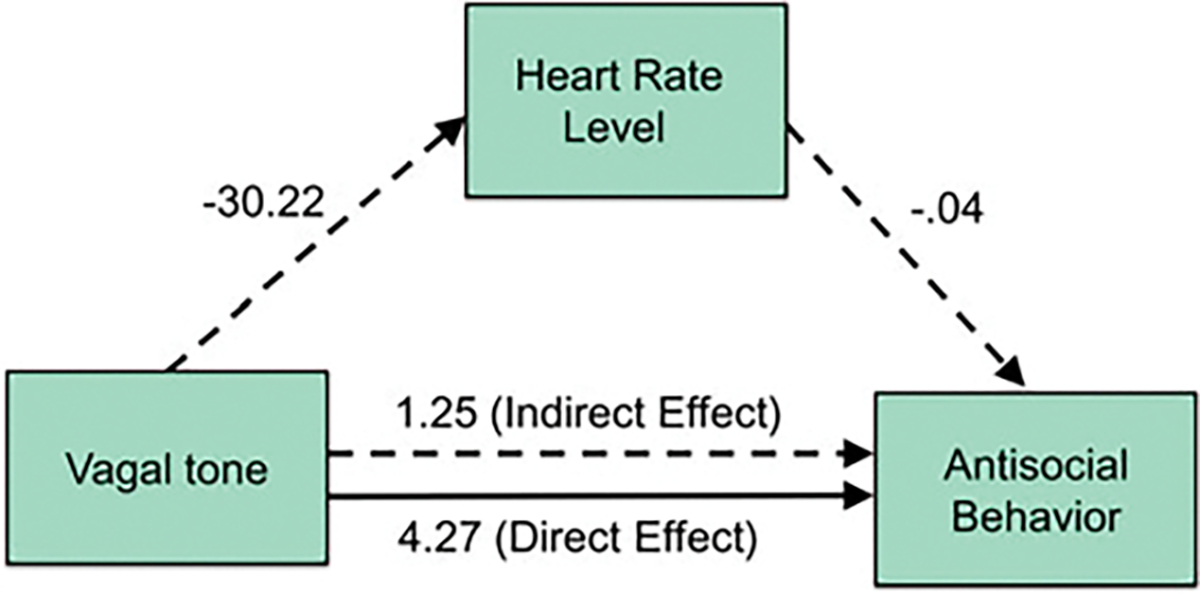
Low heart rate partly mediates the effect of increased vagal tone on increased antisocial behavior. All coefficients (unstandardized beta weights) are statistically significant, including the indirect and direct effects. Indirect paths are signified by dashed lines, the direct effect by a solid line.

Results of the test of the mediating effect of heart rate on the vagal tone – antisocial behavior relationship after controlling for covariates are given in Fig. 3. There was a significant direct effect of vagal tone on antisocial behavior, (*β* = 3.04, CI 0.052–6.02, *p* = 0.025), together with a significant indirect effect of heart rate on this relationship (*β* = 0.953, CI 0.055–2.31, *p* = 0.007). The two-step logistic regression indicated that the indirect mediation pathway accounted for 47.06% of the overall relationship between increased vagal tone and increased antisocial behavior.

## Discussion

This study set out to assess whether: (a) low HR is associated with antisocial behavior, (b) antisocial behavior is characterized by either increased or decreased vagal tone, (c) task condition moderates any observed relationship, (d) heart rate mediates any vagal tone – antisocial behavior relationship. Results indicated that (i) all antisocial groups across all conditions are characterized by lower heart rate, (ii) increased, not decreased, vagal tone characterizes antisocial behavior, and (iii) low heart rate partly mediates the relationship between increased vagal tone and increased antisocial behavior. Results indicate that increased vagal tone and reduced heart rate are relatively broad risk factors for antisocial behavior irrespective of its developmental trajectory, and are the first to implicate low heart rate as an explanatory factor in understanding vagal tone – antisocial behavior relationships. Findings provide some clarification on the basic physiological underpinnings of *why* low heart rate is associated with antisocial behavior and suggest caution for interventions aimed at increasing vagal tone to reduce antisocial behavior.

### Low heart rate

The first aim of this study was to evaluate whether low heart rate characterizes antisocial behavior. Findings confirmed the main conclusion of prior meta-analyses. The fact that all three independent samples of antisocial individuals showed lower heart rates compared to controls indicates the robustness of the findings. Effect sizes were in the small to medium range, averaging *d* = −0.41, although this is somewhat larger than the pooled effect size of −0.20 reported by Portnoy and Farrington (2015) and −0.17 reported by de Looff et al. (2022). The larger effect size in the current study using a case-control design may be partly explained by the fact that Fanti et al. (2019) similarly found a larger effect size for case-control studies (*d* = −0.33) compared to correlational studies (*d* = −0.14). Interestingly low heart rate was a significant correlate of all antisocial groupings independent of their developmental constitution, unlike our prediction that it would more characterize persistently antisocial individuals (see below on vagal tone for further elaboration).

Regarding condition, no group × condition interaction was observed, indicating that low heart rate recorded in different cognitive and affective states does not meaningfully impact findings. Consistent with this finding, de Looff et al. (2022) failed to find a moderating effect of condition on the heart rate – antisocial relationship. Regarding developmental trajectory, antisocial groups did not differ in heart rate. Overall, the current findings represent consistent support for an association between low heart rate and antisocial behavior, a relationship robust across both conditions and developmental trajectories. A future challenge lies in understanding what factors protect against future antisocial behavior in both childhood-limited and adolescent-limited antisocial individuals in the face of the putative biomarker of low heart rate.

### Increased vagal tone

The second study’s aim was to examine whether antisocial individuals are characterized by increased, or alternatively decreased, vagal tone. Results indicated that antisocial behavior was associated with increased vagal tone, with an overall effect size of *d* = 0.43 which is similar in strength to the effect size for heart rate (*d* = −0.41). While this supports the original high vagal tone hypothesis (Raine & Venables, 1984) and is consistent with some meta-analytic findings in this area (de Looff et al., 2022), it contrasts with that portion of the prior literature finding decreased vagal tone in antisocial groups. These conflicting findings are challenging and not easily resolved, but two perspectives may partly account for discrepancies.

The first lies within the framework of biosocial theory. At least three studies suggest a role of the psychosocial environment in moderating vagal tone – antisocial behavior relationships. Zhang & Gao (2015) found that high RSA was particularly associated with reactive aggression in those with high social adversity. Similarly, Scarpa, Romero, Fikretoglu, Bowser, and Wilson (1999) documented a high vagal tone – increased aggression relationship, but only in those victimized by violence. Obradović, Bush, and Boyce (2011) found that the combination of increased vagal tone and high social adversity resulted in the highest level of maladaptive behavior. Because the Pittsburg Youth Study sampled boys in public schools, the sample is weighted towards more social adversity; 66.5% of these boys had an absent parent up to the age of 13, a risk factor for antisocial behavior (Zych, Farrington, Ribeaud, & Eisner, 2021). Therefore, one reason for the striking heterogeneity in the relationship between vagal tone and antisocial behavior is that study populations may vary significantly in social status.

A second perspective concerns the form that antisocial behavior takes, and which may be particularly relevant to aggressive/violence behavior. Two prominent forms of aggression consist of reactive and proactive aggression, forms which psychophysiologically differ (Kempes, Matthys, de Vries, & van Engeland, 2005). Scarpa, Haden, and Tanaka (2010) found that while reactive aggression was associated with decreased vagal tone, proactive aggression was associated with increased vagal tone. Similarly, some studies have found that reduced vagal tone characterized reactive, but not proactive aggression (Xu, Raine, Yu, & Krieg, 2014) although others find that high vagal tone is associated longitudinally with both higher reactive and proactive aggression (Ostrov et al., 2022). While findings in this area are also somewhat inconsistent, they provide a pointer as to why different populations may differ on vagal tone, with increased vagal tone being potentially more associated with proactive aggression (see online Supplement for further elaboration and discussion on these two perspectives and the role of proactive/reactive aggression). Future studies based on demographically representative populations which range widely on social adversity levels, alongside studies evaluating both reactive and proactive aggression, may help to resolve the conflicting findings on vagal tone and antisocial behavior and test between these two perspectives.

An equally challenging issue to resolve is that all three antisocial groups showed increased vagal tone, not just the persistently antisocial group as we had hypothesized. We had based this hypothesis on the fact that this group had the most profound neurocognitive deficits compared to controls, and likely represent the group with more serious antisocial behavior (Badcock & Dragovic, 2006). At the same time, this prediction has to be tempered by the fact that childhood-only and adolescent-only also showed some forms of neurocognitive impairment, particularly in spatial immediate and delayed memory and immediate verbal memory (Raine et al., 2005). There are two possible explanations. The first is that the Pittsburgh Youth Study sample was selected to be at high risk for later crime and violence compared to unselected community samples, and as such reaching criteria for membership of any one of the antisocial groups may constitute significant antisocial behavior compared to unselected populations (Ahonen et al., 2021). Second, it is known that low heart rate is a broad risk factor for all forms of antisocial behavior, characterizing both significant and serious antisocial behavior (violent crime, sexual offenses, psychopathy, conduct disorder) and also less serious forms of antisocial behavior (oppositional behavior, school misbehavior) including crossing a pedestrian walkway on a red light (Raine, 2013). It may be that increased vagal tone, a related cardiovascular risk factor that in part determines low resting heart rate, may similarly constitute a general risk factor for all forms of ‘antisocial tendency’, regardless of seriousness level.

### Vagal passive coping response theory and task non-specificity

At a very broad level, the vagal passive coping response hypothesis (Raine & Venables, 1984) received some support in that it predicts that different antisocial groups would be characterized by increased, not decreased, vagal tone. The vagal passive coping theory of antisocial behavior hypothesizes that reduced stress reactivity results in reduced sensitivity to socializing punishments, and hence increased antisocial behavior (Raine & Venables, 1984). It was argued that in an evolutionary context, engagement of the parasympathetic nervous system in the face of impending and inescapable punishment reflects the activation of a conservation / withdrawal mechanism that disengages the individual from the impending threat, resulting in immobilization and muscular relaxation that reduces the perception of pain (Vambheim, Kyllo, Hegland, & Bystad, 2021). This passive withdrawal would reduce the impact of a socializing punishment and thus predispose to antisocial behavior. This theoretical perspective is not supported however by the lack of a group × task interaction as the theory would predict that evidence for the vagal tone – antisocial relationship would be strongest in more stressful contexts such as public speaking, but this was not observed. It has been argued however that speech tasks do not invoke passive coping (Dodo & Hashimoto, 2019), and further studies using more appropriate passive *v.* active coping tasks alongside vagal tone and antisocial measures may be better able to evaluate the vagal passive coping hypothesis.

In responding to calls for further research on antisocial grouping and conditions (de Looff et al., 2022; Lorber, 2004), we examined whether task conditions would moderate group differences in vagal tone. Contrary to our hypothesis that effects would be particularly strong in conditions evoking the most stress (speech task), relationships between vagal tone and antisocial behavior did not vary across tasks. As mentioned above, this uniformity was also not predicted by the vagal passive coping response theory (Raine & Venables, 1984), nor is it consistent with a fearlessness hypothesis of antisocial behavior (Fanti, 2018; Raine, 2002a, 2002b), both of which predict stronger effects in more stressful tasks. The current findings are however empirically consistent with the meta-regression analysis of de Looff et al. (2022) which did not observe a significant effect of task on autonomic functions that included vagal tone ( *p* = 0.46). It may be that the uniform results across tasks in the current study may be a function of this particular sample of high-risk boys and that different findings may arise in other samples. Alternatively, it may be that both vagal tone and heart rate are ‘trait’ and not ‘state’ risk factors, spanning rest, cognitive challenge, and stress states as they do in the current study. Given the current findings, we echo the calls of Lorber (2004) and de Looff et al. (2022) in emphasizing the need for further studies manipulating task conditions and antisocial sub-types, preferably within a longitudinal design, to further resolve this issue.

### Heart rate as a partial mediator of the vagal tone – antisocial relationship

The third study aim was to examine whether heart rate mediates any vagal tone – antisocial relationship. Mediation analyses documented that low heart rate partially mediated the high vagal tone – high antisocial behavior relationship. Regarding the first path from vagal tone to heart rate, there is a strong body of evidence supporting the notion that increased vagus nerve activity lowers heart rate by affecting pacemaker cells in the sinus node (Olshansky, Ricci, & Fedorowski, 2022). As such, this component of the mediation model findings appears plausible.

Regarding the second path from low heart rate to antisocial behavior, it is less clear whether low heart rate causes increased antisocial behavior, or whether it is a proxy for other variables that raise the risk of antisocial behavior. Such variables include risk-taking (Latvala et al., 2015), fearlessness (Boisvert et al., 2020; Portnoy & Farrington, 2015), stimulation-seeking (Boisvert et al., 2020; Oldehinkel, Verhulst, & Ormel, 2008; Portnoy & Farrington, 2015), and a lower likelihood of being sanctioned for antisocial behavior (Armstrong & Boutwell, 2012). Another possible mediator to be considered is the psychopathic feature of boldness which has been associated with increased vagal tone (Segarra, Poy, Branchadell, Ribes-Guardiola, & Molto, 2022). We caution that mediation was partial, accounting for 47.06% of the relationship, leaving a substantial amount of variance that could be accounted for by these other factors. Nevertheless, the current mediation analyses provide initial support for the contention that increased vagal tone may be an important source of the low heart rate – high antisocial behavior relationship (Raine & Venables, 1984).

### Intervention implications

Findings may have implications for interventions for antisocial behavior, including disruptive behavior disorders. In recent years non-invasive techniques to increase vagal tone provide an alternative to more traditional invasive forms of vagal nerve stimulation. Transcutaneous auricular vagus nerve stimulation (tVNS) is a non-invasive technique that involves electrical stimulation of the outer ear to stimulate afferent fibers of the auricular branch of the vagus nerve (ABVN) – the only branch to reach the body surface (Peuker & Filler, 2002). Stimulation activates the caudal ventrolateral region of the medulla and the dorsal motor nucleus, areas involved in the regulation of autonomic functioning. tVNS results in increased vagal tone and lower heart rate (Clancy et al., 2014), and is increasingly employed not just as a treatment for cardiovascular diseases but also for psychopathology (Wang et al., 2021; Zhu et al., 2022).

Based on some findings that low vagal tone may be associated with increased antisocial behavior, some have suggested that interventions to increase heart rate variability and thus increase vagal tone can be a potential target for antisocial behavior (Delk, Spangler, Guerra, Ly, & White, 2020). Furthermore, one recent review of tVNS concluded that this intervention can represent a potential intervention for disruptive behavior disorders (Zhu et al., 2022). We caution however that if antisocial individuals are characterized by high, and not low vagal tone, such interventions may prove counter-productive and would not be warranted. As such, any consideration of tVNS for treating disruptive behavior disorders, while potentially worthy of consideration, may first require resolution of the role of vagal tone in antisocial behaviors prior to implementation.

### Limitations and future directions

Limitations need to be acknowledged. First, this sample only consisted of males, and as such findings cannot be generalized to females. Second, while the sample was well-represented with African-American and Caucasian participants, it was limited with respect to Latino, Asian, and other ethnic representation. Third, while the assessment of antisocial behavior was longitudinal, vagal tone and heart rate were concurrently assessed; a longitudinal study separating vagal tone, heart rate, and behavior in time would provide a stronger design for testing a mediation model by establishing more clearly the temporal ordering of variables. Fourth, the CPT task induces phasic vagal reactions, and other stressful cognitive tasks might yield a different relationship to HRV (van der Molen, Somsen, & Jennings, 1996).

## Conclusions

By documenting findings in three quite different developmental forms of antisocial behavior across three different task settings, these findings add to the growing recognition that low heart rate is a relatively profound correlate of many different manifestations of antisocial behavior (Raine, 2013). Intriguingly, we found that low heart rate at age 17 was a marker for antisocial behavior in early childhood in the *absence* of antisociality at age 17 (the childhood-only group). This suggests both the trait nature of this biological correlate and also the possibility that concurrent studies of heart rate and adolescent / adult antisocial behavior may minimize the strength of this relationship when those ostensibly classified as ‘normal controls’ have an unrecorded history of early antisocial behavior.

Findings also lend credence to the notion that high – not low – vagal tone may represent a marker for antisocial behavior, although we suspect that both high and low vagal tone may be markers for antisocial behavior depending on the nature of the population under investigation. Although a number of studies have documented low vagal tone in antisocial populations, a challenge that a low vagal tone hypothesis faces is that low heart rate is reliably associated with increased vagal tone given the role of the vagus nerve in regulating heart rate (Oldehinkel et al., 2008; Olshansky et al., 2022). A reduced vagal tone – high antisocial behavior hypothesis would in contrast have to predict high, not low, heart rate as a correlate of increased antisocial behavior. This issue has not been recognized in prior studies arguing for low vagal tone in association with high antisocial behavior, and it requires further examination in future studies.

Finally, to further reconcile the conflict between the high *v.* low vagal tone hypotheses of antisocial behavior, we recommend that future studies move away from correlational designs and towards short-term experimental research that manipulates vagal tone through either tVNS (Clancy et al., 2014) or biofeedback training (Nolan et al., 2005) to better understand the nature of the relationships between heart rate, vagal tone, and antisocial behavior.

## Supplementary Material

Supplement

**Supplementary material.** The supplementary material for this article can be found at https://doi.org/10.1017/S0033291724000552

## Figures and Tables

**Table 1.** Means and s.d.s (in parentheses) for the four groups on demographics, covariates, and both heart rate and vagal tone during the three task conditions (rest, stress, and CPT)

	Controls (*N* = 139)	Childhood-Only (*N* = 51)	Adolescent-Only (*N* = 59)	Persistently Antisocial (*N* = 35)
Demographics				
Age	15.59 (0.82)	15.63 (1.11)	15.64 (0.94)	16.06 (0.93)
Ethnicity (% white)	55.4	31.4	40.7	22.9
Height (cms)	175.49 (8.63)	174.35 (7.93)	173.99 (7.97)	175.28 (7.12)
Weight (lbs)	160.56 (37.98)	165.51 (43.21)	151.84 (31.01)	159.78 (35.44)
BMI	23.60 (5.18)	24.74 (6.58)	22.62 (3.73)	23.51 (4.74)
Fitness	3.07 (1.71)	3.27 (1.82)	3.17 (1.60)	3.28 (1.89)
Caffeine	8.33 (9.04)	5.51 (6.78)	6.53 (8.11)	4.77 (6.05)
Alcohol	0.71 (4.17)	1.33 (4.72)	3.13 (7.32)	4.89 (8.94)
Nicotine	13.71 (38.75)	17.75 (32.54)	19.93 (35.85)	27.80 (35.58)
Medication (%)	0.72	0.00	0.17	0.29
Heart rate (b.p.m.)				
Rest	68.12 (10.42)	64.75 (8.77)	64.19 (9.78)	63.00 (10.13)
Stress	71.75 (10.32)	67.93 (8.51)	68.74 (9.27)	66.26 (9.71)
CPT	70.21 (10.29)	65.78 (8.76)	66.62 (9.31)	66.10 (11.07)
Vagal tone (log spectral power)				
Rest	0.270 (0.114)	0.310 (0.126)	0.327 (0.126)	0.332 (0.116)
Stress	0.273 (0.117)	0.316 (0.137)	0.307 (0.106)	0.315 (0.132)
CPT	0.267 (0.107)	0.300 (0.132)	0.300 (0.136)	0.312 (0.100)

## References

[R1] Ahonen L, Farrington DP, Pardini D, & Stouthamer-Loeber M (2021). Cohort profile: The Pittsburgh Youth Study (PYS). Journal of Developmental and Life-Course Criminology, 7(3), 481–523.

[R2] Allen JJB, Chambers AS, & Towers DN (2007). The many metrics of cardiac chronotropy: A pragmatic primer and a brief comparison of metrics. Biological Psychology, 74(2), 243–262. 10.1016/j.biopsycho.2006.08.005 17070982

[R3] Armstrong TA, & Boutwell BB (2012). Low resting heart rate and rational choice: Integrating biological correlates of crime in criminological theories. Journal of Criminal Justice, 40(1), 31–39.

[R4] Badcock JC, & Dragovic M (2006). Schizotypal personality in mature adults. Personality and Individual Differences, 40(1), 77–85.

[R5] Boisvert D, Wells J, Armstrong T, Lewis RH, Woeckener M, & Nobles MR (2020). Low resting heart rate and stalking perpetration. Journal of Interpersonal Violence, 35(11–12), 2271–2296. 29294708 10.1177/0886260517698823

[R6] Byrd AL, Vine V, Beeney JE, Scott LN, Jennings JR, & Stepp SD (2022). RSA reactivity to parent–child conflict as a predictor of dysregulated emotion and behavior in daily life. Psychological Medicine, 52(6), 1060–1068. 10.1017/S0033291720002810 PMC7908813 32799942

[R7] Clancy JA, Mary DA, Witte KK, Greenwood JP, Deuchars SA, & Deuchars J (2014). Non-invasive vagus nerve stimulation in healthy humans reduces sympathetic nerve activity. Brain Stimulation, 7(6), 871–877. 10.1016/j.brs.2014.07.031 25164906

[R8] Cohen J (1988). Statistical power analysis for the behavioral sciences (2nd ed.). Hillsdale, NJ: Lawrence Erlbaum.

[R9] Delk LA, Spangler DP, Guerra R, Ly V, & White BA (2020). Antisocial behavior: The impact of psychopathic traits, heart rate variability, and gender. Journal of Psychopathology and Behavioral Assessment, 42(4), 637–646. 10.1007/s10862-020-09813-8

[R10] de Looff PC, Cornet LJM, de Kogel CH, Fernandez-Castilla B, Embregts P, Didden R, & Nijman HLI (2022). Heart rate and skin conductance associations with physical aggression, psychopathy, antisocial personality disorder and conduct disorder: An updated meta-analysis. Neuroscience and Biobehavioral Reviews, 132, 553–582. 10.1016/j.neubiorev.2021.11.003 34774587

[R11] Dietrich A, Riese H, Sondeijker FE, Greaves-Lord K, Ormel J, Neeleman J, & Rosmalen JG (2007). Externalizing and internalizing problems in relation to autonomic function: A population-based study in preadolescents. Journal of the American Academy of Child & Adolescent Psychiatry, 46(3), 378–386. 17314724 10.1097/CHI.0b013e31802b91ea

[R12] Dodo N, & Hashimoto R (2019). Autonomic nervous system activity during a speech task. Frontiers in Neuroscience, 13, 406. 10.3389/fnins.2019.00406 31139041 PMC6518952

[R13] Fanti KA (2018). Understanding heterogeneity in conduct disorder: A review of psychophysiological studies. Neuroscience and Biobehavioral Reviews, 91, 4–20. 10.1016/j.neubiorev.2016.09.022 27693700

[R14] Fanti KA, Eisenbarth H, Goble P, Demetriou C, Kyranides MN, Goodwin D, … Cortese S (2019). Psychophysiological activity and reactivity in children and adolescents with conduct problems: A systematic review and meta-analysis. Neuroscience and Biobehavioral Reviews, 100, 98–107. 10.1016/j.neubiorev.2019.02.016 30797946

[R15] Gao Y, Huang Y, & Li X (2017). Interaction between prenatal maternal stress and autonomic arousal in predicting conduct problems and psychopathic traits in children. Journal of Psychopathology and Behavioral Assessment, 39(1), 1–14. 28286370 10.1007/s10862-016-9556-8 PMC5342840

[R16] Gianaros PJ, Van der Veen FM, & Jennings JR (2004). Regional cerebral blood flow correlates with heart period and high-frequency heart period variability during working-memory tasks: Implications for the cortical and subcortical regulation of cardiac autonomic activity. Psychophysiology, 41(4), 521–530. 15189475 10.1111/1469-8986.2004.00179.x PMC4301264

[R17] Glenn AL, & McCauley KE (2019). How biosocial research can improve interventions for antisocial behavior. Journal of Contemporary Criminal Justice, 35(1), 103–119. 10.1177/1043986218810608

[R18] Goulter N, Kimonis ER, Denson TF, & Begg DP (2019). Female primary and secondary psychopathic variants show distinct endocrine and psychophysiological profiles. Psychoneuroendocrinology, 104, 7–17. 30784904 10.1016/j.psyneuen.2019.02.011

[R19] Greenhouse SW, & Geisser S (1959). On methods in the analysis of profile data. Psychometrika, 24(2), 95–112.

[R20] Han SC, Baucom B, Timmons AC, & Margolin G (2021). A systematic review of respiratory sinus arrhythmia in romantic relationships. Family Process, 60(2), 441–456. 10.1111/famp.12644 33724463 PMC8406683

[R21] Hayes AF (2012). PROCESS: A versatile computational tool for observed variable mediation, moderation, and conditional process modeling. White Paper. http://www.afhayes.com/public/process2012.pdf

[R22] Jimenez-Camargo LA, Lochman JE, & Sellbom M (2017). Externalizing behavior in at-risk preadolescents: Relationships among effortful control, affective experiences, and autonomic psychophysiology. Journal of Psychopathology and Behavioral Assessment, 39(3), 383–395. 10.1007/s10862-017-9604-z

[R23] Kempes M, Matthys W, de Vries H, & van Engeland H (2005). Reactive and proactive aggression in children – A review of theory, findings and the relevance for child and adolescent psychiatry. European Child & Adolescent Psychiatry, 14(1), 11–19. 15756511 10.1007/s00787-005-0432-4

[R24] Laborde S, Mosley E, & Thayer JF (2017). Heart rate variability and cardiac vagal tone in psychophysiological research–recommendations for experiment planning, data analysis, and data reporting. Frontiers in Psychology, 8, 213. 10.3389/fpsyg.2017.00213 28265249 PMC5316555

[R25] Latvala A, Kuja-Halkola R, Almqvist C, Larsson H, & Lichtenstein P (2015). Resting heart rate and violent criminality: A longitudinal study of 700000 men. JAMA Psychiatry, 72, 971–978. 26351735 10.1001/jamapsychiatry.2015.1165

[R26] Loeber R, Farrington DP, Stouthamer-Loeber M, & van Kammen WB (1998). Antisocial behavior and mental health problems: Explanatory factors in childhood and adolescence. Hillsdale, NJ: Lawrence Erlbaum.

[R27] Lorber MF (2004). Psychophysiology of aggression, psychopathy, and conduct problems: A meta-analysis. Psychological Bulletin, 130(4), 531–552. 15250812 10.1037/0033-2909.130.4.531

[R28] Nolan RP, Kamath MV, Floras JS, Stanley J, Pang C, Picton P, & Young QR (2005). Heart rate variability biofeedback as a behavioral neurocardiac intervention to enhance vagal heart rate control. American Heart Journal, 149(6), 1137. e1131–1137. e1137. 10.1016/j.ahj.2005.03.015 15976804

[R29] Nuechterlein KH, Parasuraman R, & Jiang Q (1983). Visual sustained attention – image degredation produces rapid sensitivity decrement over time. Science (New York, N.Y.), 220(4594), 327–329. 10.1126/science.6836276 6836276

[R30] Obradović J, Bush NR, & Boyce WT (2011). The interactive effect of marital conflict and stress reactivity on externalizing and internalizing symptoms: The role of laboratory stressors. Development and Psychopathology, 23(1), 101–114. 21262042 10.1017/S0954579410000672

[R31] Oldehinkel AJ, Verhulst FC, & Ormel J (2008). Low heart rate: A marker of stress resilience. The TRAILS study. Biological Psychiatry, 63(12), 1141–1146. 18272139 10.1016/j.biopsych.2007.12.006

[R32] Olshansky B, Ricci F, & Fedorowski A (2022). Importance of resting heart rate: Heart rate and outcomes. Trends in Cardiovascular Medicine, 33, 8, 502–515. 10.1016/j.tcm.2022.05.006 35623552

[R33] Ortiz J, & Raine A (2004). Heart rate level and antisocial behavior in children and adolescents: A meta-analysis. Journal of the American Academy of Child and Adolescent Psychiatry, 43(2), 154–162. 14726721 10.1097/00004583-200402000-00010

[R34] Oskarsson S, Andersson A, Bertoldi BM, Latvala A, Evans B, Raine A, … Tuvblad C (in press). Lower resting heart rate as a risk factor for criminal offending among female conscripts. PLOS One. 10.1371/journal.pone.0297639 PMC10971584 38536806

[R35] Ostrov JM, Murray-Close D, Perry KJ, Blakely-McClure SJ, Perhamus GR, Mutignani LM, … Probst S (2022). The development of forms and functions of aggression during early childhood: A temperament-based approach. Development and Psychopathology, 32, 110a–110a. 10.1017/s0954579422000177 35232514

[R36] Pang KC, & Beauchaine TP (2013). Longitudinal patterns of autonomic nervous system responding to emotion evocation among children with conduct problems and/or depression. Developmental Psychobiology, 55(7), 698–706. 10.1002/dev.21065 22826111 PMC3532520

[R37] Peuker ET, & Filler TJ (2002). The nerve supply of the human auricle. Clinical Anatomy, 15(1), 35–37. 11835542 10.1002/ca.1089

[R38] Portnoy J, & Farrington DP (2015). Resting heart rate and antisocial behavior: An updated systematic review and meta-analysis. Aggression and Violent Behavior, 22, 33–45. 10.1016/j.avb.2015.02.004

[R39] Raine A (1993). The psychopathology of crime: Criminal behavior as a clinical disorder. San Diego: Academic Press.

[R40] Raine A (2002a). Annotation: The role of prefrontal deficits, low autonomic arousal, and early health factors in the development of antisocial and aggressive behavior. Journal of Child Psychology and Psychiatry, 43, 417–434. 12030589 10.1111/1469-7610.00034

[R41] Raine A (2002b). Biosocial studies of antisocial and violent behavior in children and adults: A review. Journal of Abnormal Child Psychology, 30(4), 311–326. 12108763 10.1023/a:1015754122318

[R42] Raine A (2013). The anatomy of violence: The biological roots of crime. New York: Pantheon Books.

[R43] Raine A, Moffitt TE, Caspi A, Loeber R, Stouthamer-Loeber M, & Lynam D (2005). Neurocognitive impairments in boys on the life-course persistent antisocial path. Journal of Abnormal Psychology, 114 (1), 38–49. 15709810 10.1037/0021-843X.114.1.38

[R44] Raine A, & Venables PH (1984). Tonic heart rate level, social class and antisocial behaviour in adolescents. Biological Psychology, 18(2), 123–132. 6733191 10.1016/0301-0511(84)90015-2

[R45] Sarrate-Costa C, Lila M, Comes-Fayos J, Moya-Albiol L, & Romero-Martinez A (2022). Reduced vagal tone in intimate partner violence perpetrators is partly explained by anger rumination. Current Psychology, 42, 29603–29615. 10.1007/s12144-022-03994-z.

[R46] Scarpa A, Fikretoglu D, & Luscher K (2000). Community violence exposure in a young adult sample: II. Psychophysiology and aggressive behavior. Journal of Community Psychology, 28(4), 417–425.

[R47] Scarpa A, Haden SC, & Tanaka A (2010). Being hot-tempered: Autonomic, emotional, and behavioral distinctions between childhood reactive and proactive aggression. Biological Psychology, 84(3), 488–496. 19941933 10.1016/j.biopsycho.2009.11.006

[R48] Scarpa A, Romero N, Fikretoglu D, Bowser FM, & Wilson JW (1999). Community violence exposure and aggression: Biosocial interactions. Paper presented at the meeting of the American Society of Criminology.

[R49] Segarra P, Poy R, Branchadell V, Ribes-Guardiola P, & Molto J (2022). Psychopathy and heart rate variability: A new physiological marker for the adaptive features of boldness. Personality Disorders-Theory Research and Treatment, 13(5), 557–562. 10.1037/per0000573 35511573

[R50] Vambheim SM, Kyllo TM, Hegland S, & Bystad M (2021). Relaxation techniques as an intervention for chronic pain: A systematic review of randomized controlled trials. Heliyon, 7(8). 10.1016/j.heliyon.2021.e07837 PMC8405991 34485731

[R51] van der Molen MW, Somsen RJ, & Jennings JR (1996). Does the heart know what the ears hear? A heart rate analysis of auditory selective attention. Psychophysiology, 33(5), 547–554. 10.1111/j.1469-8986.1996.tb02431.x 8854742

[R52] Wagner NJ, & Waller R (2020). Leveraging parasympathetic nervous system activity to study risk for psychopathology: The special case of callous-unemotional traits. Neuroscience and Biobehavioral Reviews, 118, 175–185. 10.1016/j.neubiorev.2020.07.029 32745477

[R53] Wang Y, Li SY, Wang D, Wu MZ, He JK, Zhang JL, … Rong PJ (2021). Transcutaneous auricular vagus nerve stimulation: From concept to application. Neuroscience Bulletin, 37(6), 853–862. 10.1007/s12264-020-00619-y 33355897 PMC8192665

[R54] Weber EJ, Molenaar PC, & Van der Molen MW (1992). A nonstationarity test for the spectral analysis of physiological time series with an application to respiratory sinus arrhythmia. Psychophysiology, 29(1), 55–62. 1609027 10.1111/j.1469-8986.1992.tb02011.x

[R55] Xu YY, Raine A, Yu LD, & Krieg A (2014). Resting heart rate, vagal tone, and reactive and proactive aggression in Chinese children. Journal of Abnormal Child Psychology, 42(3), 501–514. 23959546 10.1007/s10802-013-9792-2

[R56] Zhang W, & Gao Y (2015). Interactive effects of social adversity and respiratory sinus arrhythmia activity on reactive and proactive aggression. Psychophysiology, 52(10), 1343–1350. 26175181 10.1111/psyp.12473

[R57] Zhu SY, Zhang XL, Zhou MH, Kendrick KM, & Zhao WH (2022). Therapeutic applications of transcutaneous auricular vagus nerve stimulation with potential for application in neurodevelopmental or other pediatric disorders. Frontiers in Endocrinology, 13, 1000758, Article 1000758. 10.3389/fendo.2022.1000758 36313768 PMC9596914

[R58] Zych I, Farrington DP, Ribeaud D, & Eisner MP (2021). Childhood explanatory factors for adolescent offending: A cross-national comparison based on official records in London, Pittsburgh, and Zurich. Journal of Developmental and Life-Course Criminology, 7(3), 308–330.

