# Supplementary material for: Increased cardiac vagal tone in childhood-only, adolescent-only, and persistently antisocial teenagers: the mediating role of low heart rate
Source: Psychol Med. Author manuscript; Available in PMC 2026 Jan 16. (PMC12809886; doi:10.1017/S0033291724000552)
Supplement: Supplement [file NIHMS2131043-supplement-Supplement.docx]

**Online Supplement:**

Increased cardiac vagal tone in childhood-limited, adolescent-limited, and life-course persistent teenagers: The mediating role of low heart rate.

**Cluster Analysis Producing the Three Antisocial and One Non-Antisocial Groups**

*Delinquency seriousness classification groups* (*age 7-17 years)*

Delinquency measures were obtained from parents (using an extended version of the CBCL, Achenbach & Edelbrock, 1979), teachers (using an extended version of the Teacher Report Form, Edelbrock & Achenbach, 1984), and boys (using the Self-Report Delinquency scale, Elliott, Ageton, Huizinga, Knowles, & Canter, 1983). Full methodological details of the classification are provided in Loeber et al. (1998). Assessments were taken every six months from ages 7 to 11 years, and every year thereafter up to 17 years. At each stage, subjects were classified into six levels of delinquency seriousness based on an extension of the four-stage delinquency seriousness classification (see Loeber et al., 1998). Most behaviors in the classification system were represented by more than one question and more than one respondent. Classifications were based on the most serious act reported by any of the informants.

Items from the three informant sources were weighted for seriousness using severity ratings developed by Wolfgang, Figlio, Tracy, & Singer (2002) and used to construct delinquency seriousness categories for each of the 14 assessments as follows: no delinquency (scored 0), minor delinquency at home, (e.g. stealing from parents, scored 1), minor delinquency other (e.g. shoplifting, scored 2), moderately serious delinquency (e.g. gang fighting, scored 3), serious delinquency (e.g. car theft, scored 4), and serious-violent delinquency (e.g. attack to seriously hurt or kill, scored 5). The mean 1-year test-retest reliability for the classification system was 0.41 (p < .001).

*Cluster analysis*

Inspection of the dendrogram indicated two large clusters (antisocial and non-antisocial groups), as well as four clusters with N sizes of 156, 57, 68, and 44. Similarly, inspection of the plot of fusion coefficients indicated a large jump in values at the two-cluster level as well as a jump at the four-cluster level. Antisocial behavior data for these four clusters across ages 7-17 years are shown in Figure 1. Groups differed significantly on delinquency scores at all ages, *F*(3,321) > 24.4, *p* < .0001. It can be seen that one group (Controls, N = 156) remain stably low on antisocial behavior from age 7 to 17. A second group (AL: on the Adolescent-Limited path, N = 68) start off at exactly the same level as the Controls, but progress to significant levels of antisocial behavior by late adolescence. A third group (CL: Childhood-Limited path, N = 57) start off with high levels of antisocial behavior up to age 11 but then decline. A fourth group (LCP: on the Life-Course Persistent path, N = 44) start off high and shown even higher levels of antisocial behavior during late adolescence.

Figure S1. Group mean delinquency seriousness scores for the four clusters from ages 7 to 17 years.

**Two Perspectives on Discrepant Findings on Vagal Tone and Antisocial Behavior**

As outlined in the manuscript, the literature on vagal tone and antisocial behavior is inconsistent, with some studies reporting increased vagal tone and some reporting decreased vagal tone. In an effort to further understand these conflicting findings, we provided two very provisional perspectives:

1. the relationship may be dependent on social adversity, with studies on populations coming from more stressful / adverse backgrounds tending to produce high vagal tone – high antisocial behavior findings.
2. Increased vagal tone may characterize more proactive, planful forms of aggression, while decreased vagal tone may characterize more reactive, impulsive aggression.

In this context, one reviewer asked whether the antisocial/delinquent behavior in this study is particularly proactive, thus supporting the second perspective. We had measured proactive and reactive aggression in this sample at age 16 when heart rate and vagal tone were examined using the Reactive-Proactive Aggression Questionnaire (RPQ) and full findings have been previously reported (Raine *et al.*, 2006). Those evaluated in the current paper score as follows: proactive aggression: M = 2.89, SD = 3.60; reactive aggression: M = 7.24, SD = 4.20. Although scores are higher on reactive compared to proactive aggression, this is typical of all populations studied to date on this instrument and reflects the notion that reactive aggression is more common, and potentially less pathological, than proactive aggression. Without further empirical data to go on, we suspect that our sample is not necessarily over-represented with proactive aggression relative to reactive aggression, although certainly we believe that the sample perpetrates more significant proactive forms of aggression than most community samples.

We attempted to evaluate further whether proactive aggression plays a key role in the findings by repeated the primary analysis after entering proactive aggression as a covariate. If proactive aggression plays a role at some level in the findings of increased vagal tone in the antisocial groups, it might be expected that the significant difference between controls and antisocial groups would be attenuated. Before covariation, the main effect of group was significant, F=3.45, df (3, 280), p=.017, indicating increased vagal tone in the antisocial groups. After entering *proactive* aggression scores as a covariate, the main effect of group was no longer significant, F=1.58, df (3, 276), p=.194.

This result on the face of it would provide some support for the second perspective that suggests some role of proactive aggression. However, we repeated the analyses entering *reactive* aggression as covariate, and this again rendered the main results non-significant, F=2.08, df (3, 276), p=.103, although the extent of change was not quite as strong as for proactive aggression.

We further examined the reviewer’s suggestion by correlating proactive, reactive, and total aggression with the averaged measure of vagal tone. Results showed that increased vagal tone was associated with *both* proactive (r = .16, p = .007, N = 292) and reactive aggression (r = .17, p = .004, N = 292), as well as with total aggression (r = .18, p = .002, N = 292).

Overall, we conclude:

1. that overall our findings do not provide significant support for the second perspective
2. the fact that increased vagal tone also significantly characterizes two different forms of aggression further adds to the notion that antisocial behavior (whether “childhood limited”, “adolescent limited”, “life-course” persistent, reactive aggression, proactive aggression) is associated, at least in this sample, with increased vagal tone.

We reiterate that the conflicting findings in the literature on vagal tone and antisocial behavior are challenging and not easily resolved, and furthermore a clearer resolution probably go well beyond the two alternatives suggested here. For example, another interpretation of the conflicting findings that has been put forward is that increased vagal tone more characterize increased antisocial behavior in community samples, whereas decreased vagal tone characterizes antisocial behavior in clinic samples (Pang and Beauchaine, 2013). A further possibility is that developmental processes may play a role, with the low vagal tone - antisocial relationship being more likely to be obtained in child samples, whereas the high vagal tone – antisocial relationship may be observed more in later adolescence and adulthood.

References

**Pang, K. C. & Beauchaine, T. P.** (2013). Longitudinal Patterns of Autonomic Nervous System Responding to Emotion Evocation Among Children With Conduct Problems and/or Depression. *Developmental Psychobiology* **55**, 698-706.

**Raine, A., Dodge, K., Loeber, R., Gatzke-Kopp, L., Lynam, D., Reynolds, C., Stouthamer-Loeber, M. & Liu, J. H.** (2006). The reactive-proactive aggression questionnaire: Differential correlates of reactive and proactive aggression in adolescent boys. *Aggressive Behavior* **32**, 159-171.
